# Evaluation of the Anti-Plasmodial potential of *Calotropis procera* Latex in mice infected with *Plasmodium berghei*

**DOI:** 10.1016/j.mex.2021.101528

**Published:** 2021-09-24

**Authors:** Johnson Adejoh, Lukman A. Alli, Michael P. Okoh

**Affiliations:** Department of Medical Biochemistry, Faculty of Basic Medical Sciences, College of Health Sciences, University of Abuja, P.M.B 117 FCT, Abuja, Nigeria

**Keywords:** Anti-Plasmodial, *Calotropis procera*, Phytochemical, Malaria and *Plasmodium berghei*

## Abstract

Current artemisinin-based combination therapy (ACT) regimen for the treatment of malaria is effective, but cases of malaria parasite's resistance to existing antimalarial drugs is worrisome. This necessitates the development of new, safe, effective and affordable chemotherapy. We describe protocols for each step involved in the Anti-Plasmodial evaluation of *Calotropis procera* latex in mice infected with *Plasmodium berghei*. The protocols include: (1) determination of the chemical/ phytochemical constituents of *Calotropis procera* latex, (2) determination of the acute toxicity/ median lethal dose (LD_50_) and therapeutic dose of the plant latex, *in vivo*, and (3) *in vivo* determination of the Anti-Plasmodial potential of *Calotropis procera* latex in mice infected with *Plasmodium berghei*. We likewise describe our methodology for direct quantitation of percentage yield of the extract of *Calotropis procera* latex, and the statistical methodology for assessment of toxicity and efficacy in evaluating the Anti-Plasmodial activity of the plant.

• Multi-step pipeline for the extraction of the bioactive constituents of the plant latex using 0.2M phosphate buffer (pH 7.0) and cold acetone.
• Detailed protocols for the determination of acute toxicity/ median lethal dose (LD_50_) and calculation of therapeutic dose for intraperitoneal injection to achieve effective dose levels.
• Determination of the phytochemical constituents using standard procedures, and *in vivo* efficacy against *Plasmodium berghei* using methodology to directly quantify the parasite level after treatment.

## Graphical abstract

**Figure:**
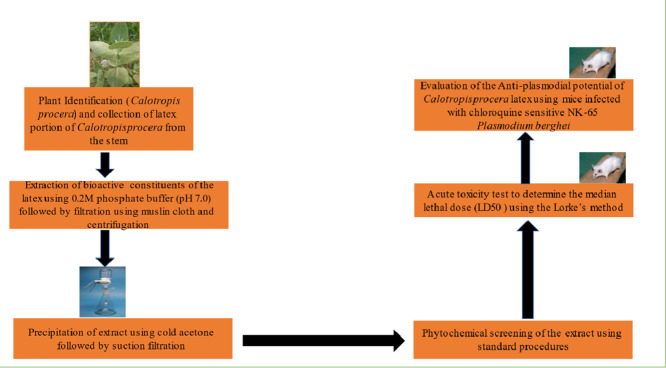

Specifications table

**Table:** 

Subject Area:	Medical Biochemistry and Phytomedicine
More specific subject area:	Anti-plasmodial evaluation
Method name:	**Anti-plasmodial evaluation of *Calotropis procera* latex in mice**
Name and references of original method:	*N/A (See Reference section)*
Resource availability:	All consumables and equipment required are as described in our recent publication, Adejoh et al. (2021).

***List of materials***

1. Plant – *Calotropis procera* (Aiton) Dryand
2. Plant part used - Latex
3. Animal – Swiss albino mice of both sexes, weighing between 17 to 25g and aged 4 to 6 weeks
4. Parasite – *Plasmodium berghei* NK 65 Chloroquine sensitive strain
5. Extraction Materials: 0.2M phosphate buffer (pH 7.0), Theodor Svedberg analytical centrifuge (at 10,000 rpm for 15 min) and rotary shaker.
6. Precipitation – Cold acetone, suction filtration apparatus
7. LD_50_ determination – Weighing balance, Hematocrit reader for measuring Packed Cell Volume (PCV), Randox glucose diagnostic kit (for measuring blood glucose level), Agape kits (for evaluation of Total protein, Albumin, Alanine aminotransferase (ALT), Aspartate Transaminase (AST), Alkaline phosphatase (ALP)).
8. Drug – Chloroquine (used for positive control)
9. Normal saline (used for negative control)
10. 0.05M phosphate buffer (pH 7.6) (used for suspension of air-dried extract for storage)
11. Monosodium phosphate (Acid) (Used for buffer preparation)
12. Disodium phosphate (conjugate base) (used for buffer preparation)
13. Distilled water
14. Phosphoric acid (to make the buffer pH more acidic)
15. Sodium hydroxide (to make the buffer pH more alkaline)
16. pH Meter
17. Glassware
18. Hot plate with stirring bar
19. Measuring cylinder
20. Beaker
21. Petri dishes
22. Test tubes
23. Test tube rack

## Protocol

### Sample collection

*Calotropis procera* plant sample was collected at the University of Abuja Main Campus, FCT-Abuja, Nigeria. It was identified and authenticated for use at the Department of Medicinal Plant Research and Traditional Medicine of National Institute of Pharmaceutical Research and Development (NIPRD), Abuja, Nigeria. A voucher number “NIPRD/H/7110” was assigned and kept at the NIPRD herbarium for future reference. The latex samples used for the study were collected from the stem of the plant using a 10 ml syringe and placed in a clean sample bottle, stored in refrigerator at temperature of -20°C to prevent further denaturation and loss of proteases before the commencement of extraction. The plant was confirmed present on http://www.theplantlist.org.

### Extraction of the bioactive/ chemical constituents of the plant latex

The bioactive/ chemical constituents of the plant latex were extracted following the experimental procedures developed by Abdullahi et al. [[1], [2]] with slight modification in terms of volume. The steps involved are outlined below:

• Weigh 25g of the crude latex obtained from the plant stem using analytical graded weighing balance;
• Transfer the weighed latex into a conical flask;
• Measure 50 ml of 0.2M phosphate buffer solution (pH 7.0), pour into the conical flask containing the latex and stir continuously for 20 min;
• Filter the latex-phosphate buffer mixture (in step 3) using muslin cloth to remove cell debris;
• Transfer the filtered latex-phosphate buffer mixture into test tubes and centrifuge (using Theodor Svedberg analytical centrifuge) for 15 min at 10,000 rpm to obtain a clear filtrate;
• Separate the clear filtrate into a beaker for acetone precipitation.

### Preparation of 0.2M phosphate buffer (pH 7.0) solution for the extraction

An initial stock solution of 1 molar concentration was prepared following the steps below:

• Using the Henderson-Hasselbach equation, the amount of acid and base needed were calculated. This was simplified by preparation of 1 Liter of the buffer.
• A pKa value of 6.9 that is closest to the pH (7.0) of the desired buffer was selected. pH = pKa + log ([Base]/[Acid]) ratio of [Base]/[Acid] = 1.096
  The molarity of the buffer is the sum of the molarities of the acid and conjugate base or the sum of [Acid] + [Base]. For a 1 M buffer (selected for ease of calculation), [Acid] + [Base] = 1.
  [Base] = 1 - [Acid].
  This was substituted into the ratio and the amount of acid was determined as thus:
  [Base] = 0.523 moles/L. [Base] = 1 - [Acid], so [Acid] = 0.477 moles/L.
• The solution was prepared by mixing 0.477 moles of monosodium phosphate and 0.523 moles of disodium phosphate in a little less than a liter of water.
• The 0.477 moles of the monosodium phosphate were prepared by dissolving 57.24g of the powdered salt in 1L of water. The mass was determined using the relationship: Mass = Amount x Molar Mass (i.e., Mass = 0.477 × 120 = 57.24g). In a similar way, 0.523 moles of the disodium phosphate were prepared by dissolving 74.27g of the powdered salt in 1L of water. The same relationship as above was also used. (That is, Mass = 0.523 × 142 = 74.27g).
• The pH of the resulting solution from step 3 was checked using a pH meter and adjusted as necessary using phosphoric acid or sodium hydroxide.
• Upon reaching the desired pH, water was added to bring the total volume of phosphoric acid buffer to 1 L.
• From the stock solution, the desired buffer of 0.2M solution (pH 7.0) used for the extraction was prepared using the dilution principle. The principle is represented mathematically as thus:
  $$C_{1}V_{1}=C_{2}V_{2}$$

Where:

C_1_ = Initial concentration/ molarity of the stock solution;

C_2_ = Final concentration/ molarity of the diluted solution;

V_1_ = Initial volume of the stock;

V_2_ = Final volume of the diluted solution;

### Precipitation of bioactive/ chemical constituents of the plant latex (acetone precipitation)

The bioactive/ chemical constituents of the plant latex were precipitated using cold acetone followed by suction filtration. This was based on the modified protocols earlier developed by Abdullahi et al. [1] with adjusted changes in terms of volume ratio of cold acetone to latex extract and the duration of precipitation. The detail steps are:

• 50 ml of crude latex extract (clear filtrate) obtained after centrifugation was transferred into a conical flask;
• 25 ml of cold acetone was added into the conical flask containing the clear filtrate and stirred continuously for 20 min;
• After 20 min of stirring, the conical flask was set up in a vacuum pump for suction filtration to separate the precipitate from the mixture;
• The precipitate obtained in step 3 was placed in a petri dish and allowed for air drying;
• The air-dried acetone powder was suspended in 0.05M phosphate buffer (pH 7.6) and stored at 4°C;
• The percentage yield of the dried extract is calculated using the relationship:

  % yield of dried extract = weight of dried extract / weight of wet crude latex X 100.

• An aliquot portion of this extract were weighed and dissolved in distilled water for preparation of extract solution for phytochemical screening and appropriate doses for acute toxicity determination and Anti-Plasmodial activity on each day of the experiment.

### Determination of the extract chemical constituents/ phytochemical screening

The extract was screened for phytochemical constituents, using standard procedures as described by Sahira and Catherine [[11], [13]] with slight modification in terms of solvent concentration and volume. Each of the phytochemical constituents were screened using different solvents as described herein.

## Test for alkaloids (Mayer's Test)

0.5g of the extract was weighed and transferred into a watch glass, thereafter, 1 mL of 5% hydrochloric acid (HCl) and 2 drops of Mayer's reagents were added. Formation of white creamy precipitate will indicate the presence of alkaloids [6].

## Test for flavonoids (Ammonia test)

5 ml of 1M ammonia solution were added to a portion of the extract sample followed by the addition of 1ml of 5% H_2_SO_4_. The formation of yellow coloration will indicate a positive test for flavonoids [8,17].

## Test for saponins (Froth Test)

The extracts were diluted with distilled water to 20 mL and were shaken in a graduated cylinder for 15 min. Formation of a 2 cm layer of foam around the cylinder will indicate the presence of saponins [7].

## Test for tannins (Braymer's Test)

0.5g of the dried, powdered sample was boiled in 20 mL of distilled water in a test tube. The resultant material was then filtered. 2 drops of 0.1% ferric chloride solution were added to the mixture. Formation of green-blue coloration is a positive test for tannins [17].

## Test for terpenes (Salkowski Test)

5 mL of the filtrate was mixed with 2 ml of chloroform,-3 mL of 5% H_2_SO_4_ was carefully added to form a layer. Formation of golden-yellow layer (at the bottom) will indicate the presence of terpenes [14,17].

## Test for phenolic compound (Ferric Chloride test)

The extract (50 mg) is dissolved in 5 mL of distilled water. Few drops of neutral 5% ferric chloride solution were added. The formation of a dark green colour will indicate the presence of phenolic compound [[15], [16]].

## Test for cardenolides (Libermann-Burchard's test)

The extract (50 mg) is dissolved in of 2 mL acetic anhydride. To this mixture, 1 or 2 drops of concentrated sulphuric acid are added slowly along the sides of the test tube. An array of colour change will show the presence of cardenolides [10].

## Test for resin (Acetic anhydride test)

1 mL of plant extract was dissolved in acetic anhydride solution, followed by addition of 1 mL concentrated H_2_SO_4_. Color change from orange solution to yellow will indicate the presence of resins [8,14].

### Acute toxicity/ therapeutic dose determination of the extract of *Calotropis procera* latex

The doses were selected based on the results obtained for the acute toxicity/ median lethal dose (LD_50_) of the extract. The acute toxicity/ median lethal dose of the extract was determined following the standard procedures developed by Lorke's [9] with slight modification in terms of extract doses of phase II. The tests were carried out in two phases; In phase I, nine female mice, weighing 20 to 25g were equally divided into three groups (A, B and C). The extracts were administered intraperitoneally to the groups at graded doses of 10 mg/kg, 100 mg/kg, and 1000 mg/kg body weight, respectively and observed for 24 h. A second phase was carried out following the death of all mice administered with 1000 mg/kg body weight by ranging the dosage below 1000 mg/kg.

In phase II, three female mice, weighing 20–25g were equally divided into three groups (D, E and F) and each group received extract doses of 250 mg/kg, 500 mg/kg, and 750 mg/kg body weight, respectively.

The LD_50_ was determined by finding the geometric mean of the highest dose that produced non-lethal effect (i.e., D_o_ = 500 mg/kg) and the lowest dose that gave lethal effect (i.e., D_1_ = 750 mg/kg). LD_50_ = Geometric mean of D_o_ and D_1_. Where Do is the highest dose that produced non-lethal effect, D1 is the lowest dose that gave lethal effect. The result of the geometric mean of these doses gave 745 mg/kg (which is the acute toxicity/ median lethal dose of the extract).

The therapeutic doses of the extract were determined from the median lethal dose by simply taking one-tenth of the median lethal dose (i.e., therapeutic dose = 1/10 of 745 mg/kg = 74.5 mg/kg ∼ 75 mg/kg as the highest dose for the Anti-Plasmodial determination).

### Preparation of plasmodium parasite inoculum by serial passage

The plasmodium parasite inoculum was prepared by serial passage following the protocols developed by Alli et al. [3].

• Two mice, weighing 25g each were inoculated with cultured *Plasmodium berghei* NK 65 chloroquine sensitive strain and allowed to stay for 5–8 day of post inoculation;
• Percentage parasitemia level of each mice were determined using the relationship: % Parasitiemia = (Number of parasitized Red Blood Cell (RBC)/ (Total number of RBC) x 100;
• A donor mouse with a parasitemia level of 20% was sacrificed and blood drawn into a Heparinised syringe and diluted with normal saline;
• Infection was initiated by intraperitoneally injecting 0.2 ml of the parasite preparation from a donor mouse to healthy mice;
• Parasitemia level of each infected mice were monitored by microscopic Giemsa-stained thin blood smears;
• The number of parasitized erythrocytes in about 5–10 fields was counted twice and the average computed to give the parasitemia of each mouse.

### Evaluation of the Anti-Plasmodial activity of extract of *Calotropis procera* latex

The antimalarial property of the extract of *Calotropis procera* latex was evaluated by examination of its suppressive, prophylactic and curative potentials, *in-vivo,* using the method of Bulus et al*.,* [4] with slight modification in terms of number mice used and dosage.

### Suppressive test (Peter's Test)

The Peter's 4-day suppressive test against chloroquine sensitive *Plasmodium berghei* (NK 65) infection in mice was employed [3] and modified to align with experimental design. The procedures include:

• Malaria parasite inoculums were prepared by collecting blood samples from mice (donor mice) infected with *Plasmodium berghei;*
• The blood samples were diluted with normal saline buffer (pH 7.0), such that 0.2 mL contained 1 × 10^7^ of the parasites;
• Thirty (30) mice were divided into 5 groups of 6 mice each;
• The mice were infected with the parasites by inoculating them with 0.2 mL of the prepared blood solution in step 2;
• Treatment commenced two hours after inoculation and continued daily for four consecutive days (D_1_-D_4_);
• Group 1, 2 and 3 were administered with graded extract doses of 25mg, 50mg and 75mg extract/kg body weight/day intraperitoneal, respectively;
• Group 4 (Negative control group) was administered with 0.2 ml normal saline;
• Group 5 (chloroquine treated group) was administered with 5 mg chloroquine/kg body weight/day intraperitoneally;
• On the fifth day (D_5_), blood samples were collected from the caudal vein of each mouse on to a clean slide and smeared evenly;
• The thin blood film was fixed on the slide, using methanol and was followed by staining with Giemsa stain (dye) after which the number of the parasitized cells were determined microscopically [5].
• Average parasite count and the percentage suppression evaluated by using the following equation:

**Average Suppression** = APC – APT / APT X 100
***APC****= Average Parasitaemia in the Negative Control,****APT****= Average Parasitaemia in the Test group*

### Curative test (Rane Test)

Evaluation of the curative potential of *Calotropis procera* latex extract against established infection was carried out as described by Ryley and Peters [12] with slight adjustment in line with the experimental design for this study. The procedures include:

• Thirty (30) mice were all inoculated with *Plasmodium* parasite as described above (for suppressive test) and left untreated until the fifth day (D_5_) post inoculation to initiate infection;
• The mice were weighed and randomized into five groups of six mice each;
• Mice in Group 1, 2 and 3 were administered with graded extract doses of 25 mg, 50 mg and 75 mg extract/kg body weight/day intraperitoneal, respectively;
• Group 4 (Negative control group) was administered 0.2 ml normal saline buffer (pH 7.0);
• Group 5 (chloroquine treated group) was administered with 5mg chloroquine/kg body weight/day intraperitoneally for four days (D_5_-D_8_);
• On Day-7 (after 48 h of treatment) and Day-9 (after 96 h of treatment), each mouse was tail bled and a thin blood film was made on a microscope slide;
• The films were stained with Giemsa stain and examined using a microscope (BS-2030T (500C) model) to monitor the parasitemia level.
• Average parasite count and percentage cure for the extract was evaluated using the above equation for percentage suppression.

### Prophylactic test (Repository Test)

Evaluation of the prophylactic potential of *Calotropis procera* latex extract against *Plasmodium berghei* infection in mice was carried out as described by Alli et al. [3] with slight modification in line with the experimental design for this study.

• Thirty (30) mice were weighed and randomized into five groups of 6 mice each, and were treated for four days (D_1_-D_4_);
• Group 1, 2 and 3 were administered with graded extract doses of 25mg, 50mg and 75mg extract/kg body weight/day intraperitoneal, respectively;
• Group 4 (Negative control group) was administered 0.2ml normal saline buffer (pH 7.0);
• Group 5 (chloroquine treated group) was administered with 5mg chloroquine/kg body weight/day intraperitoneally, also, for four days;
• On the fifth day, the treated mice were inoculated with the parasites and then left for four days (Day_5_-Day_8_) before the presence of the parasites was determined microscopically;
• On Day-8, each mouse was tail-bled and a thin blood film was made on a microscope slide;
• The films were stained with Giemsa stain and examined microscopically to determine the parasitemia level;
• Average parasite count and percentage prophylaxis for the extract was evaluated using the above equation for average suppression.

### Data analysis

Statistical analyses were performed using JMP v.14 software (SAS Institute, Cary, NC, USA). One-way ANOVA with Dunnett's post-test was used to test the significance of differences between the treatment groups. Statistical significance was set at *P* < 0.05 for both mean parasitaemia count and mean weight of the parasitized mice Table 1.

**Table 1:** Reagent preparation for phytochemical screening.

Reagents/ Solutions		Composition
1	Mayer's reagent	**Solution A:** 1.358g mercuric chloride + 60 mL distilled water **Solution B:** 5g potassium iodide + 10 mL distilled water **Working solution:** solution A + solution B + distilled water to make final volume 100mL
2	Ferric Chloride solution	1. Take 67.5 gm of ferric chloride and make paste with 5 mL Con. HCl 2. Now add slowly distilled water to make up the solution up to the mark in volumetric flask.
3	Ammonia solution	1. Measure 71.0 ml of conc. ammonia solution. 2. Transfer this to1 L volumetric flak. 3. Add distilled water to this and make up the solution up to the mark in volumetric flask.
4	Acetic acid solution	1. Measure 58.0 mL of glacial acetic acid. 2. Transfer this in 1 L volumetric flak. 3. Add distilled water to this and make up the solution up to the mark in volumetric flask.

Shaikh and Patil, 2020.

## Declaration of Competing Interest

We declare no conflict of interest.
